# Auxin inhibits chlorophyll accumulation through ARF7-IAA14-mediated repression of chlorophyll biosynthesis genes in *Arabidopsis*

**DOI:** 10.3389/fpls.2023.1172059

**Published:** 2023-04-20

**Authors:** Wei-Gui Luo, Qi-Wen Liang, Yi Su, Chao Huang, Bei-Xin Mo, Yu Yu, Lang-Tao Xiao

**Affiliations:** ^1^ College of Physics and Optoelectronic Engineering, Shenzhen University, Shenzhen, China; ^2^ Guangdong Provincial Key Laboratory for Plant Epigenetics, Longhua Bioindustry and Innovation Research Institute, College of Life Sciences and Oceanography, Shenzhen University, Shenzhen, China; ^3^ College of Bioscience and Biotechnology, Hunan Agricultural University, Changsha, China

**Keywords:** auxin, Yucca, chlorophyll biosynthesis, AUXIN RESPONSE FACTOR (ARF), transcription repression

## Abstract

Auxin is a well-known important phytohormone in plant that plays vital roles in almost every development process throughout plant lifecycle. However, the effect of auxin on the metabolism of chlorophyll, one of the most important pigments involved in the photosynthesis, was intertwined and the underlying mechanism remained to be explored. Here, we found the auxin-defective *yuc2 yuc6* double mutant displayed dark-green leaf color with higher chlorophyll content than wildtype, suggesting a negative regulatory role of auxin in chlorophyll biosynthesis. The chloroplast number and structure in mesophyll cells were altered and the photosynthetic efficiency was improved in *yuc2 yuc6*. In addition, the chlorophyll level was significantly improved during seedling de-etiolation in *yuc2 yuc6* mutant, and decreased dramatically under IAA treatment, confirming the inhibitory role of auxin in chlorophyll biosynthesis. The analyses of gene expression in mature leaves and de-etiolation seedlings suggested that auxin suppressed the expression of many chlorophyll biosynthesis genes, especially *PROTOCHLOROPHYLLIDE OXIDOREDUCTASE A* (*PORA*) and *GENOMES UNCOUPLED 5* (*GUN5*). Yeast-one-hybrid and luciferase assays demonstrated that the AUXIN RESPONSE FACTOR 2 (ARF2) and ARF7 bind to the promoter of *PORA* and *GUN5* to suppress their expression with the help of INDOLE-3-ACETIC ACID14 (IAA14). Collectively, our research explicitly unraveled the direct inhibitory role of auxin in chlorophyll biosynthesis, and provided new insight into the interplay between auxin signaling and chlorophyll metabolism.

## Introduction

Chlorophyll is a vital pigment in photosynthesis through playing critical roles in light energy harvesting and charge separation in photosystem I (PSI) and photosystem II (PSII) in green plant (Ohashi et al., 2010; Kim et al., 2020). During seed germination in the dark, skotomorphogenesis occurs to develop etioplasts. Upon exposure to light, photomorphogenesis program takes place, including the transition of etioplasts into fully functional chloroplasts accompanied with chlorophyll biosynthesis (Mysliwa-Kurdziel et al., 2012; Jedynak and Malec, 2015). As a branch of the tetrapyrrole biosynthetic pathway, the process is quite complex composing of multiple enzyme-catalyzed reactions and involving numerous enzymes and genes (Ruediger and Grimm, 2006; Scheer, 2006; Tanaka et al., 2011; Fiedor et al., 2019). The Glu-tRNA is firstly converted to 5-aminolevulinic acid (ALA), followed by several enzymatic steps to process protoporphyrinogen IX (Proto IX), the common precursor of haem and chlorophyll (Oborník and Green, 2005; Kobayashi and Masuda, 2016). Then Mg^2+^ was inserted into Proto IX to form chlorophyll *a* (Kobayashi et al., 2008), and subsequently came into chlorophyll cycle, the conversation between chlorophyll *a* and chlorophyll *b* (Ruediger, 2006; Masuda, 2008). To coincide with the construction of the photosynthetic machinery, the metabolism of chlorophyll and the intermediates are strictly regulated and organized.

Auxin is a versatile plant hormone that regulates numerous physiological and developmental processes throughout the lifecycle of plants, such as cell division and expansion, embryogenesis, floral organ development, vascular system patterning and leaf senescence (Woodward and Bartel, 2005; Aloni et al., 2006; Cecchetti et al., 2008; Sauer et al., 2013). The biosynthesis and signaling pathways of auxin have been well established. The primary natural auxin in plants is indole-3-acetic acid (IAA), which is mainly synthesized through a TRYPTOPHAN AMINOTRANSFERASE OF ARABIDOPSIS (TAA)/YUCCA (YUC) two-step tryptophan-dependent pathway (Zhao et al., 2001; Vanneste and Friml, 2009; Mashiguchi et al., 2011; Won et al., 2011). With a low amount of auxin, the Auxin/Indole-3-Acetic Acids (Aux/IAAs) protein interacts with AUXIN RESPONSE FACTORS (ARFs), which act as either transcriptional activators or repressors of auxin-responsive genes to regulate its activity (Guilfoyle et al., 2005; Guilfoyle and Hagen, 2007; Roosjen et al., 2018; Cance et al., 2022). When auxin accumulates to a certain level, TRANSPORT INHIBITOR RESISTANT1 (TIR1)/AUXIN SIGNALING F-BOX (AFB), a subunit of the SCF ubiquitin ligase complex, perceives auxin and promotes the ubiquitin-mediated degradation of Aux/IAAs to activate the transcriptional activities of ARFs (Woodward and Bartel, 2005; Calderon Villalobos et al., 2012; Strader and Zhao, 2016).

Several studies have illustrated that ARFs contribute to the accumulation of chlorophyll by modulating the expression of genes related to chlorophyll metabolism, but the effects of ARFs are controversial. For example, a dark-green phenotype was observed in the tomato fruit overexpressing *SlARF10*, which positively regulated the expression of *GOLDEN1-LIKE* (*SlGLK1*), *PROTOCHLOROPHYLLIDE OXIDOREDUCTASE* (*POR*), *CHLOROPHYLL BINDING PROTEIN* (*CBP1*, and *CBP2*) (Yuan et al., 2018). Overexpression of SlARF6A, which directly binds to the promoters of *SlGLK1*, *CHLOROPHYLL A/B BINDING PROTEIN* (*CAB*), and *RIBULOSE BISPHOSPHATE CARBOXYLASE SMALL CHAIN* (*RbcS*) genes to promote their expression, resulted in increased chlorophyll contents in the tomato fruits and leaves (Yuan et al., 2019). In contrast, SlARF4 negatively regulates chlorophyll accumulation and starch biosynthesis in tomato fruit through repressing transcription of *SlGLK1* (Sagar et al., 2013). The opposite effects of auxin in chlorophyll accumulation have also been reported in both algae and plants. For example, the addition of synthesized auxin naphthalene-acetic-acid (NAA) leads to a positive correlation between the increased chlorophyll content and biomass in *Pleurochrysis portfolio* and *Chlorella sorokiniana* (Hunt et al., 2011). Indole-3-acetic acid (IAA) increases chlorophyll *a* and *b* content in *Solanum lycopersicum* and *Cinnamomum camphora* (Dao et al., 2018; Zhou et al., 2020). By controlling the concentration of synthesized auxin 2,4-D, the production of chlorophyll in *Chlorella vulgaris* and *Spirulina platensis* can be easily changed (Saygideger and Okkay, 2008), which have been applied into producing herbicides. On the other hand, auxin negatively regulates chlorophyll biosynthesis in Arabidopsis root *via* the function of ARF7, ARF19 and the repressor protein IAA14 (Kobayashi et al., 2012). In addition, it is reported that accumulation of protochlorophyllide (Pchlide), the precursor of chlorophyll, was increased in the etiolated seedlings of auxin-defective mutants *iaa3* and *iaa7* accompanied with an up-regulated transcription levels of tetrapyrrole biosynthetic genes *HEMA1* and *CHLH* in etiolated seedlings (Hedtke et al., 2012). However, the molecular mechanisms underlying the auxin-mediated regulation of gene expression in chlorophyll biosynthesis still remain largely unknown so far.

In this study, we found the auxin-defective *yuc2 yuc6* double mutant exhibited greener leaf color compared to wildtype. Further investigation showed that *YUC2* and *YUC6* regulated the chlorophyll accumulation, chloroplast development and photosynthesis activity *via* auxin. In addition, auxin negatively regulated the expression of chlorophyll biosynthesis genes through ARF2, ARF7 and IAA14. Collectively, our study revealed the regulatory mechanism of auxin in chlorophyll biogenesis, which would provide new insights into the improvement of photosynthesis in plants.

## Materials and methods

### Plant materials and growth conditions

*Arabidopsis thaliana* wild-type (accession Columbia-0) and T-DNA insertion lines of *yuc2* (SALK_030199) and *yuc6* (SALK_093708) single mutant lines in the Columbia-0 background were used in this study. *yuc2 yuc6* double mutant was obtained by crossing. Genotype was identified as descripted on T-DNA Primer Design website (http://signal.salk.edu/tdnaprimers.2.html).

The 2 kb genomic sequence upstream of transcription start site of *PORA* and *GUN5* were amplified and cloned into the *pCAMBIA1301* vector with *HindIII* and *NcoI* restriction sites to produce *proPORA::GUS* and *proGUN5::GUS* plasmids. The plasmids were introduced into the *Agrobacterium tumefaciens* strain GV3101 and introduced into plants by the floral dip method (Zhang et al., 2006). Primer sequences were listed on the **Supplemental Table S2**.

*Arabidopsis thaliana* seeds were surface-sterilized in 70% ethanol for 30 sec and treated with 1% sodium hypochlorite solution for 5 min, followed by washing with sterile distilled water 3-5 times. The surface-sterilized seeds were vernalized at 4°C for 3 days, and then were grown on solid medium (1/2 Murashige and Skoog medium, 1% sucrose, and 0.8% agar, pH 5.8) at 22°C under long photoperiod conditions (16-h-light/8-h-dark). After Two-week-old seedlings were transplanted to soil and grown under the same condition.

For de-etiolation assay, seeds were germinated on 1/2 MS solid medium (1/2 MS media supplied without sucrose), with or without 1 μM IAA, at 22°C under continuous dark. 5-day-old etiolated seedlings were transferred to light to accelerate greening. Seedlings exposed to light for 0 h, 6 h and 12 h were collected for chlorophyll determination or RNA extraction.

### Chlorophyll content determination

Chlorophyll determination was carried out according to the spectrophotometric methods described by Lichtenthaler and Buschmann (Lichtenthaler and Buschmann, 2005).

### Microscopic analyses

Fresh leaves were collected and fixed in a pre-cooled 4% glutaraldehyde solution overnight at 4°C. Next, the samples were washed three times with the 50 mM PBS (pH 7.0) and postfixed in 2% osmic acid for 2-3 h. Thereafter, sections were dehydrated in an acetone series and infiltrated and embedded in propylene oxide-resin. Finally, samples were cut into slices and photographed with scanning transmission electron microscope JEM-1230.

### Photosynthetic rate and chlorophyll fluorescence measurements

The photosynthetic rate was determined by a portable photosynthesizer (LI-6400, LI-COR). The instrument was calibrated and setup according to the operation instructions. Related parameters were recorded when keeping the leaves *in-situ* upon the leaf chamber.

Chlorophyll fluorescence parameters were measured with pulse-amplitude modulation fluorometer (PAM-2500; Walz) according to the manufacturer’s instructions. Prior to the measurement, plants were dark-adapted for 30 min to set all PSII centers to the open state. In the fluorescence induction experiments, data were collected in a logarithmic time series between 50 ms and 300 ms after the onset of strong actinic light. The maximum quantum yield of PSII ([Fm-Fo]/Fm) was calculated from the minimum fluorescence yield (Fo) and the maximum fluorescence yield (Fm). In the fluorescence decay experiments, data were collected between 50 ms and 300 ms following a single saturation flash.

### GUS staining

Tissues were harvested and fixed in pre-cooled 90% acetone on ice for 30 min and then washed with GUS washing buffer (50mM NaH_2_PO_4_ pH7.2, 50mM Na_2_HPO_4_ pH 7.2, 10 mM Na_2_·EDTA, 0.1% Triton-X100, 0.5 mM K_3_Fe(CN)_6_, and 0.5mM K_4_Fe(CN)_6_) for three times. Then the samples were transferred to GUS staining buffer (GUS washing buffer with 2mM X-Gluc) and vacuumized for 3-5 times. Then the samples were incubated at 37°C for overnight. The stained tissues were photographed using an Olympus BH-2 microscope equipped with an Olympus DP12 digital camera after chlorophyll removal in 70% ethanol.

### RNA isolation and qRT-PCR analysis

Total RNAs were extracted from leaves or de-etiolation seedlings were extracted by using a TransZol Up regeant (Transgene, Cat# ET111-01). The genomic DNA was removed by incubation with DNase I (Takara, Cat# 2270A) for 30 min at 37°C. After phenol/chloroform extraction, the purified RNA was used to synthesize cDNA with RevertAid Reverse Transcription Kit (Thermo Scientific, Cat#K1691). qRT-PCR was performed in a BIORAD qPCR instrument using ChamQ SYBR qPCR Master Mix (Vazyme, Cat#Q311). Expression values were calculated by the 2^-△△CT^ method using *ACTIN2* as a reference gene and analyzed by Prism7 software (Graphpad). Three biological and three technical replicates were performed for each assay. Primers used were listed in **Supplemental Table S3**.

### Yeast-one-hybrid assay

The cis-elements of chlorophyll biosynthesis genes were analyzed using the database PlantPAN 3.0 (http://plantpan.itps.ncku.edu.tw/) (Chow et al., 2019). Upstream genomic fragments of the chlorophyll biosynthesis genes containing *AuxREs* were cloned and inserted into *pAbAi* vector with the HindIII-XhoI site to generate *pBait-AbAi* vectors. *p53-AbAi* was served as the negative control. The *pBait-AbAi* and *p53-AbAi* vectors were transformed into *Saccharomyces cerevisiae* strain Y1HGold according to the Matchmaker^®^ Gold Yeast One-Hybrid System User Manual from Clontech to generate *pBait-AbAi* strains. The entire coding fragments of *ARF2* and *ARF7* was cloned into the EcoRI-BamHI sites of the *pGADT7* prey vector and transformed into *pBait-AbAi* strains respectively. The Yeast One-hybrid Assay was conducted as previously described (Zhang et al., 2018).

### Luciferase reporter assay

The 2 kb genomic fragment upstream of the chlorophyll biosynthesis gene were cloned into the *pGreenII 0800-LUC* vector with BamHI site to generate *LUC* reporter plasmids. The full-length CDSs of *ARF2*, *ARF7* and *IAA14* were cloned into the *pGreenII 62-SK* vector with BamHI-HindIII sites to generate assay effectors, and the *pGreenII 62-SK* empty vector was used as the negative control. These plasmids were transformed into Agrobacterium strain GV3101 (*pSoup-P19*) and infiltrated into *Nicotiana benthamiana* leaves (Popescu et al., 2007). After incubation for 72 h, the abaxial epidermis of infiltrated leaves was smeared with 1 mM beetle luciferin (Promega, cat#E1603) and the illumination signal was captured under Lumazone pylon2048B *In Vivo* Imaging System.

## Results

### Knock out of *YUCCA2* and *YUCCA6* affects chlorophyll content

**Figure 1:**
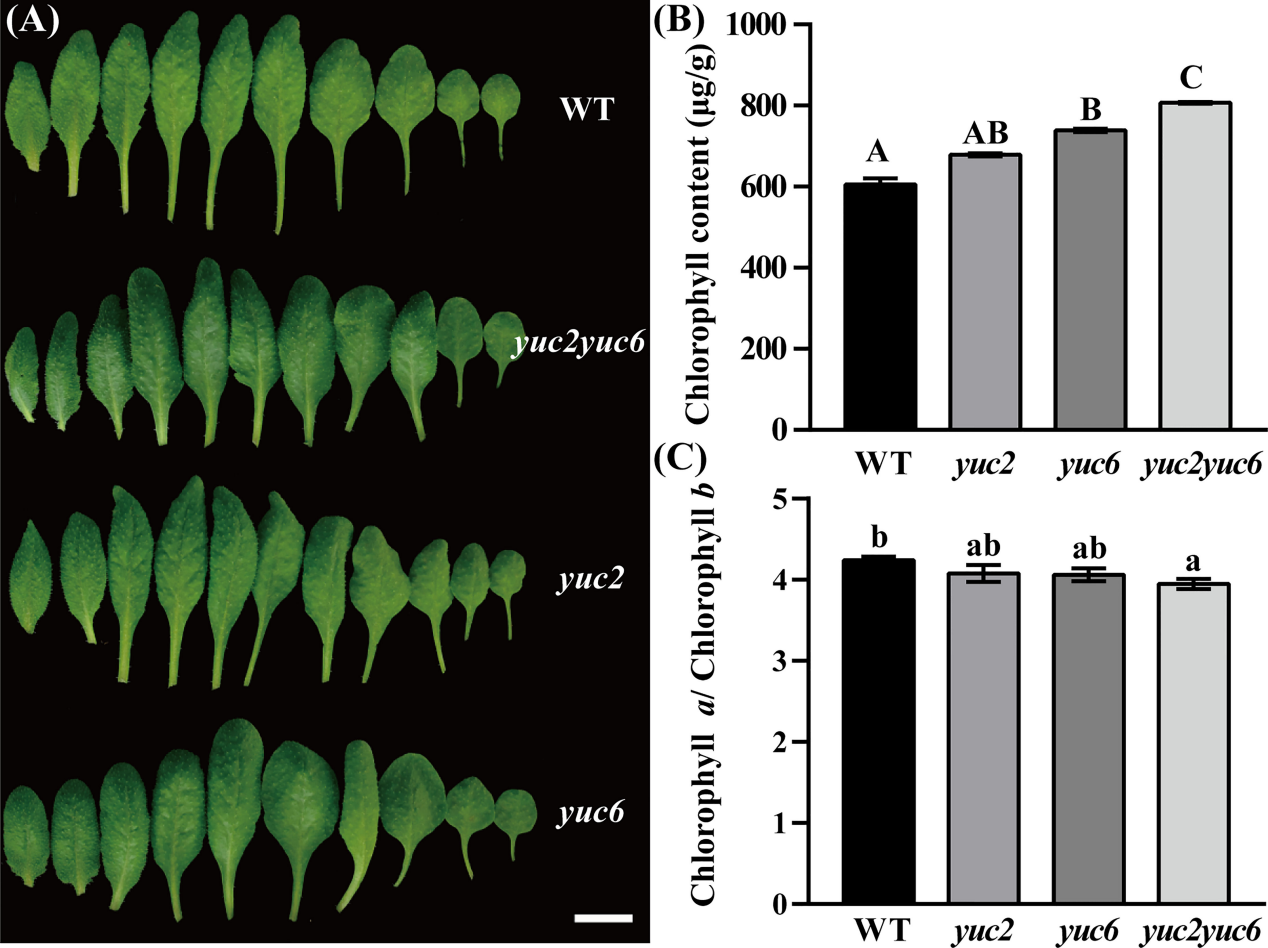
Phenotype of *yuc2 yuc6* leaves. **(A)** Leaves of 4-weeks-old plant, bar = 1 cm. **(B)** Chlorophyll content in WT and mutants. **(C)** Ratio of chlorophyll *a* and *b* in WT and mutants. The capitals represent *p* value < 0.01, and the lowercases represent *p* value < 0.05.

*YUCCA2* (*YUC2*) and *YUCCA6* (*YUC6*) are two essential genes for auxin biosynthesis and participate in male fertility regulation in Arabidopsis, *yuc2 yuc6* double mutant was male sterile (Cheng et al., 2006; Cheng and Zhao, 2007; Yao et al., 2018). However, the leaf phenotype of the double mutant has not been reported yet. We found that at the vegetative stage, the rosette leaves of *yuc6* single and *yuc2 yuc6* double mutants are noticeable greener than that of WT (wildtype), while *yuc2* is similar to WT (**Figure 1A**, **Figure S1**). To verify these observations, the total chlorophyll content as well as the chlorophyll *a* and *b* were determined in WT and each mutant. As expected, the total chlorophyll level was highest in *yuc2 yuc6*, followed by *yuc6* and *yuc2*, and was lowest in WT (**Figure 1B**). As for the ratio of chlorophyll *a* and *b*, the order was just the opposite with WT highest and *yuc2 yuc6* mutant lowest (**Figure 1C**). These quantitative results were consistent to the gradation of leave colors, indicating that *YUC2* and *YUC6* acted redundantly in regulating chlorophyll accumulation, and *YUC6* played a dominant role in this process. Knockout of *YUC2* and *YUC6* would result in decreasing auxin level undoubtedly, as they are two essential genes for auxin biosynthesis. We checked auxin level by inducing the *DR5::GUS* reporter, a commonly used auxin-reporter line for approximate assessment of auxin concentration and distribution in plants, into WT and *yuc2 yuc6*, respectively. As show in **Figure S2**, auxin level decreased dramatically in *yuc2 yuc6* seedlings and leaves compared to that in WT. Taken together, the increased chlorophyll content and decreased amount of auxin implied that auxin play a negative role in chlorophyll biosynthesis.

### *YUC2* and *YUC6* are required for the proper development of chloroplasts

**Figure 2:**
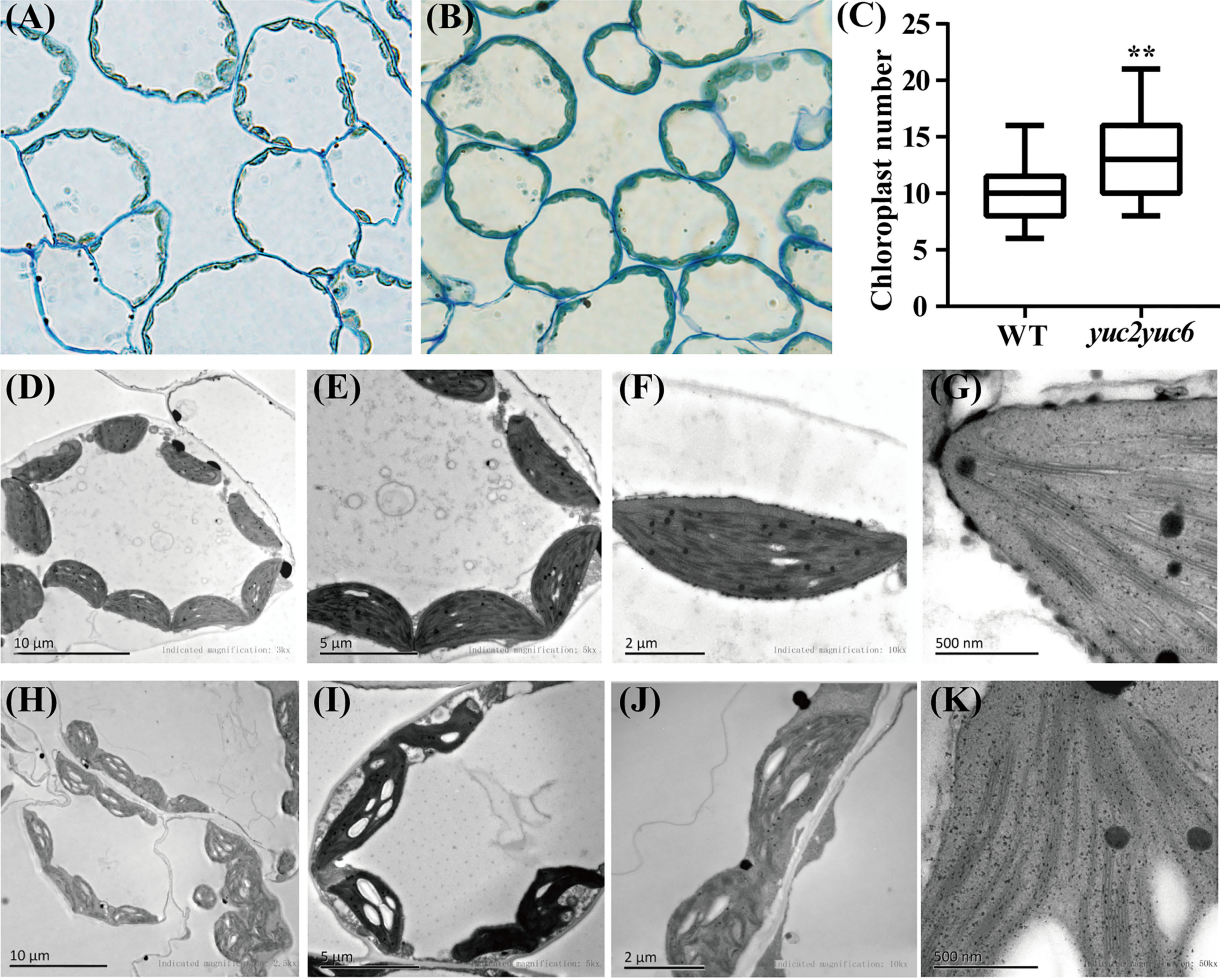
Chloroplast morphology of mesophyll cells in rosette leaves of WT and *yuc2 yuc6*. **(A)** Mesophyll cells of WT. **(B)** Mesophyll cells of *yuc2 yuc6*. **(C)** Chloroplast numbers of mesophyll cells. **(D-G)** Chloroplast ultrastructure of WT. **(H-K)** Chloroplast ultrastructure of *yuc2 yuc6*. ** represents *p* value < 0.01.

The biosynthesis of chlorophyll occurs coordinately with biogenesis of chloroplasts from proplastids, so the effect of *YUC2* and *YUC6* on chloroplast development deserves to be explored. The chloroplast morphology of mesophyll cells was analyzed in rosette leaves of WT and *yuc2 yuc6* double mutant. Apparently, the average chloroplast number per mesophyll cells in *yuc2 yuc6* mutant was larger than that in WT (**Figures 2A-C**). In addition, the chloroplasts were uniformly spindle-shaped and distributing evenly around the inner side of the cell membrane in WT, whereas the chloroplasts of *yuc2 yuc6* mutants were irregularly shaped and arranged unevenly (**Figures 2D-E, H-I**), implying that mutations in *YUC2* and *YUC6* may result in developmental defects of chloroplasts. Moreover, many plastids developed starch grains in addition to thylakoid membranes in *yuc2 yuc6* (**Figures 2E-G, I-K**), reflecting a high chloroplast-developing activity in the mutant. Collectively, these results suggested that *YUC2* and *YUC6* were essential for chloroplast development.

### Photochemical efficiency is improved in *yuc2 yuc6*

**Figure 3:**
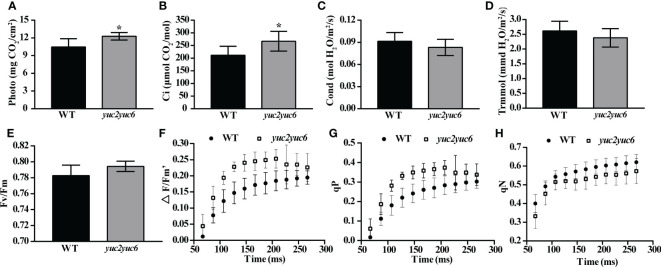
Improved photosynthesis in *yuc2 yuc6*. **(A)** Photosynthetic rate. **(B)** Intercellular CO_2_ concentration. **(C)** Stomatal conductance. **(D)** Transpiration rate. **(E)** Maximum quantum yield Fv/Fm ratio. **(F)** Real-time photosynthetic efficiency. **(G)** Photochemical quenching coefficient. **(H)** Non-photochemical quenching coefficient. * represents *p* value <0.05.

As chlorophyll is an important pigment involved in photosynthesis and chloroplasts are the specific organelles that photosynthesis occurs in, the alteration of chlorophyll content and chloroplast structure will inevitably affect photosynthesis efficiency. Therefore, the photosynthetic parameters were measured in four-week-old WT and *yuc2 yuc6* mutant. The photosynthetic rate and the intercellular CO_2_ concentration were significantly higher in *yuc2 yuc6* than those in WT (**Figures 3A, B**), whereas the stomatal conductance and transpiration rate were slightly lower in *yuc2 yuc6* than those in WT (**Figures 3C, D**), indicating a stronger photochemical efficiency in *yuc2 yuc6* mutant than that in WT. To further assess involvement of *YUC2* and *YUC6* in the repression of photosynthesis, the chlorophyll fluorescence parameters (index to indicate photosynthesis ability) were determined by chlorophyll fluorescence instrument PAM-2500 in WT and *yuc2 yuc6* mutant. The maximum quantum yield Fv/Fm ratio, which reflects the potential maximum photosynthetic capacity, was higher in *yuc2 yuc6* mutant than that in WT, although the difference was moderate (**Figure 3E**). In addition, the actual quantum yield ΔF/Fm’ ratio in *yuc2 yuc6* mutant was higher than that in WT at each time point (**Figure 3F**), indicating that the efficiency of photosynthetic electron transport was improved in *yuc2 yuc6.* There are two complementary parameters qP and qN. qP is the photochemical quenching coefficient, which is the fluorescence quenching caused by photosynthesis, representing the photosynthetic activity of the plants. qN is a non-photochemical quenching coefficient, which is the fluorescence quenching caused by heat dissipation, indicating the plant ability to dissipate excess light energy into heat (photoprotective ability). Higher qP and lower qN corresponding to higher photosynthetic activity were detected in *yuc2 yuc6* (**Figures 3G, H**). Taken together, these results confirmed that the photosynthesis was improved in *yuc2 yuc6.*

### Auxin inhibits chlorophyll accumulation in seedlings during de-etiolation

**Figure 4:**
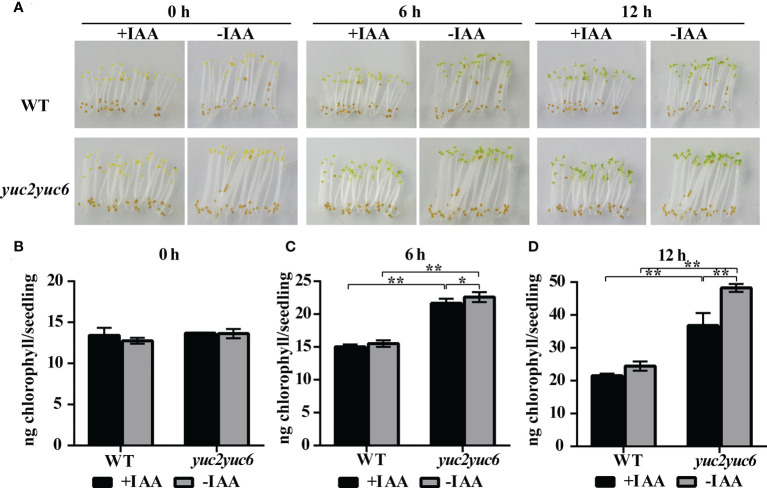
Auxin inhibits chlorophyll accumulation during de-etiolation. **(A)** Etiolated seedlings exposed to light under IAA treatment. **(B-D)** Chlorophyll content at each time point during de-etiolation. ‘+IAA’ represents seedlings treated with 1 μM IAA, ‘-IAA’ represents seedlings within no IAA treatment, * represents *p* value <0.05, ** represents *p* value <0.01.

Chlorophyll accumulation is one of the most important events during greening of photosynthetic organs and requires the light signaling. To examine whether *YUC2* and *YUC6* affect chlorophyll accumulation in seedlings during de-etiolation *via* auxin, chlorophyll contents were measured every 6 hours after light exposure in the etiolated WT and *yuc2 yuc6* seedlings, which were grown on 1/2 MS plates with 1 μM IAA (+IAA) or without (-IAA) in dark for 5 days. Apparently, without IAA treatment, the cotyledons of *yuc2 yuc6* mutant turn green earlier with considerably higher chlorophyll amount than that in WT after exposure to light (**Figure 4**). However, the inhibition of chlorophyll accumulation was observed in both WT and *yuc2 yuc6* mutant seedlings when exogenous IAA was applied, and the difference was more significant in *yuc2 yuc6* than WT (**Figures 4B-D**). These data suggested that both endogenous and exogenous auxin acts as a negative regulator of chlorophyll accumulation in cotyledons during de-etiolation.

### *YUC2* and *YUC6* inhibit the expression of chlorophyll biosynthesis genes *via* auxin

**Figure 5:**
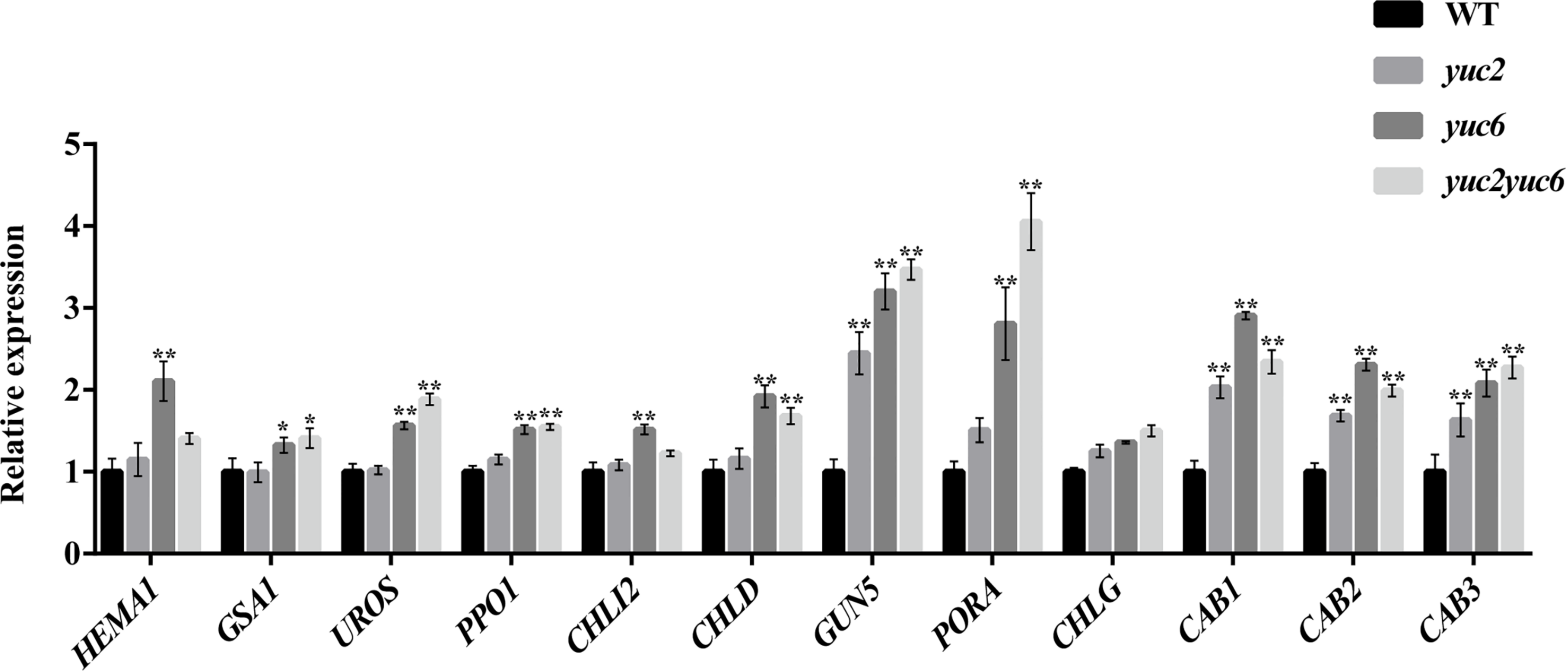
Chlorophyll gene expression of rosette leaves. * represents *p* value <0.05, ** represents *p* value <0.01.

To gain more insight into the mechanism of *YUC*-mediated chlorophyll metabolism, we examined the expression profiles of several chlorophyll biosynthesis genes in the leaves of four-week-old WT, *yuc2*, *yuc6* and *yuc2 yuc6* mutants by qRT-PCR. As shown in **Figure 5**, all 12 examined genes showed a generally higher transcript levels in *yuc* single and/or double mutants than that in WT. A strong upregulation of *PORA* and *GUN5* was observed in *yuc2 yuc6*, indicating they were negatively regulated by *YUC2* and *YUC6*.

**Figure 6:**
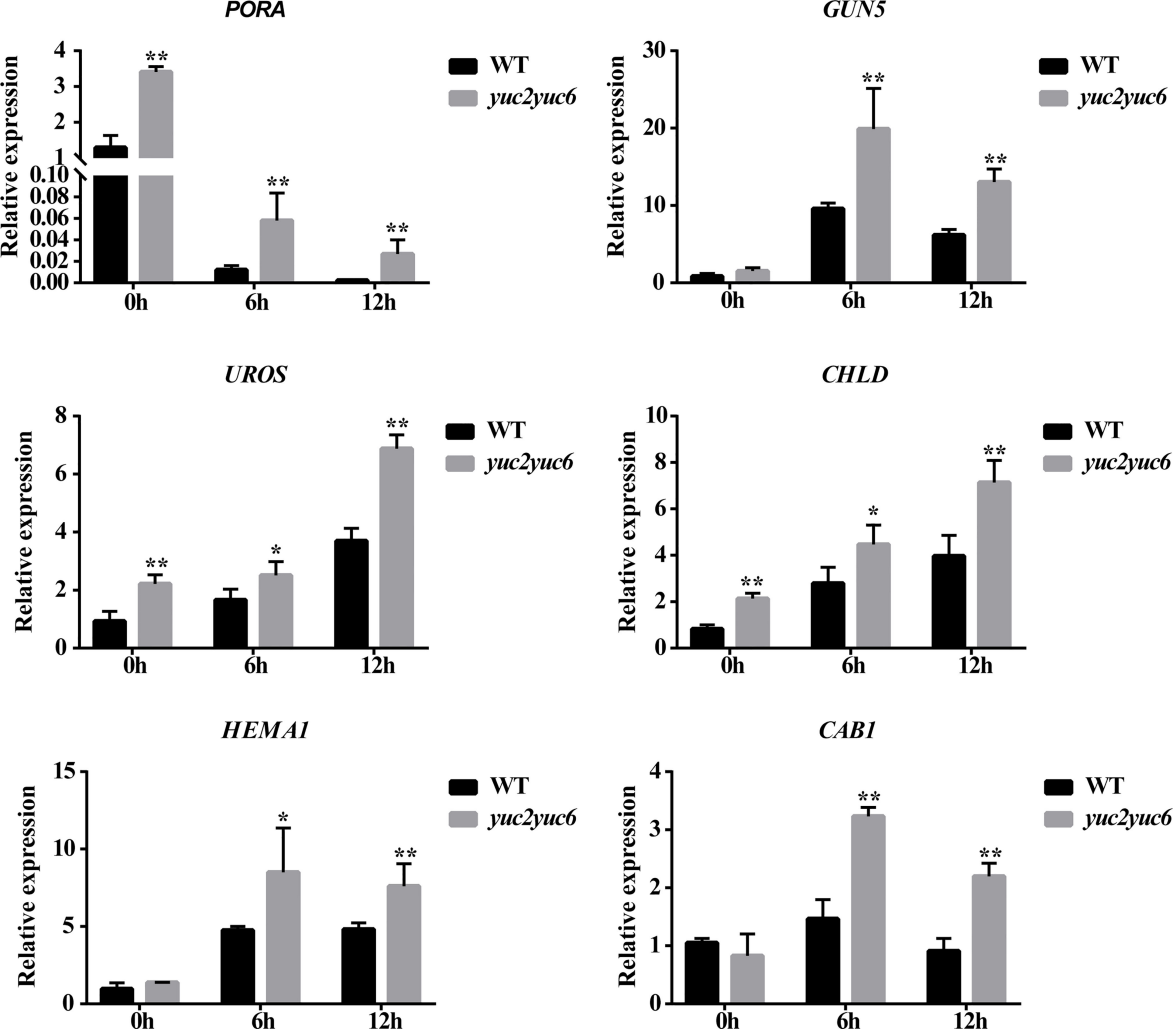
Chlorophyll gene expression of seedlings during de-etiolation. * represents *p* value <0.05, ** represents *p* value <0.01.

To further evaluate the influence of *YUC2* and *YUC6* on the expression of chlorophyll biosynthesis genes, we analyzed the transcript levels of some chlorophyll genes in WT and *yuc2 yuc6* seedlings during de-etiolation. The expression of all the genes was induced by light in both WT and *yuc2 yuc6* except for *PORA* (**Figure 6**). *PORA* encodes a NADPH: protochlorophyllide oxidoreductase and is primarily expressed at early development stage-during etiolation, germination and greening. It has been reported that *PORA* mRNA rapidly disappears after illumination (Armstrong et al., 1995; Holtorf et al., 1995; Paddock et al., 2012), which is consistent with our results. Although the expression levels some genes were comparable between WT and *yuc2 yuc6* before de-etiolation, all of these genes showed a higher expression level in *yuc2 yuc6* than WT after light exposure. The chlorophyll gene expression pattern is consistent with the phenotypes and chlorophyll showed ahead, suggesting that the suppression of *YUC2* and *YUC6* on chlorophyll accumulation was resulted from the down-regulated expression of biosynthesis genes.

**Figure 7:**
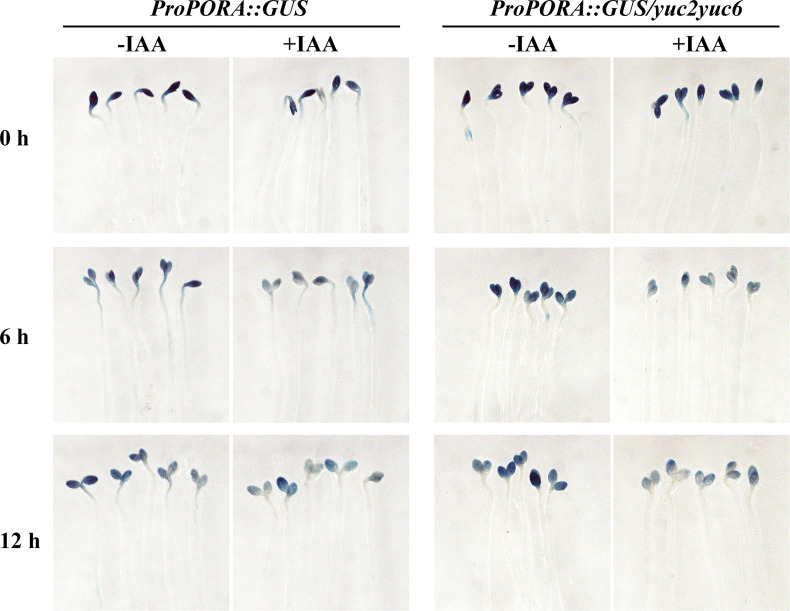
Auxin suppresses the expression of *GUS* driven by *PORA* promoter.

To further verify the auxin inhibition on the expression of chlorophyll biosynthesis gene expression, promoter of *PORA* (*proPORA*) and *GUN5* (*proGUN5*) driving β-glucuronidase (GUS) reporter were introduced into WT and *yuc2 yuc6* respectively. Homozygosis seeds of T3 transplants were plated as mentioned in de-etiolation assay and seedlings were stained with GUS solution after light exposure (**Figure 7**). For *proPORA::GUS* transgene plants without IAA treatment, *GUS* expression was attenuated with increasing illumination time in both WT and *yuc2 yuc6*, consistently with the quantification of gene expression showed ahead. As expected, the GUS expression was decreased significantly in both WT and *yuc2 yuc6* under IAA treatment, implying that IAA suppressed the transcriptional activity of *PORA* promoter. Similarly, the expression of *GUS* driven by *GUN5* promoter was also decreased under IAA treatment (**Figure S3**), indicating the repression role of auxin on *GUN5* expression.

### ARFs bind to chlorophyll biosynthesis gene promoters and suppress their transcription activity

The suppression of IAA on the transcriptional activities of *proPORA* and *proGUN5* suggested the interaction between ARFs and these promoters. We surveyed the *Auxin Response Elements* (*AuxREs*), which could be bound by ARFs, in the promoter of chlorophyll biosynthesis genes. 12 genes promoter were predicted to contain one or several *AuxREs* in their promoter regions (**Table S1**). Among the ARFs, ARF7 and ARF19 were reported to play redundant roles in chlorophyll accumulation in detached root (Kobayashi et al., 2012). We also observed that *arf2* mutant exhibited dark green leaf color that similar to *yuc2 yuc6* (data not shown), implying that ARF2 may be involved in the regulation of chlorophyll biosynthesis. Therefore, ARF2 and ARF7 were selected as the candidates to investigate whether ARFs regulated chlorophyll genes directly. In addition, the expression levels of *ARFs* genes in rosette leaves were examined in WT and *yuc2 yuc6*, respectively. Most of the *ARFs* genes,; including *ARF2* and *ARF7*, were down-regulated in *yuc2 yuc6* (**Figure S4A**). The expression levels of *ARF2* and *ARF7* in seedlings during de-etiolation were also checked in WT and *yuc2 yuc6*. *ARF2* showed lower transcript level at 0 h in *yuc2 yuc6* compared to that in WT, whereas *ARF7* level was lower at 6 h in *yuc2 yuc6* (**Figure S4B**).

**Figure 8:**
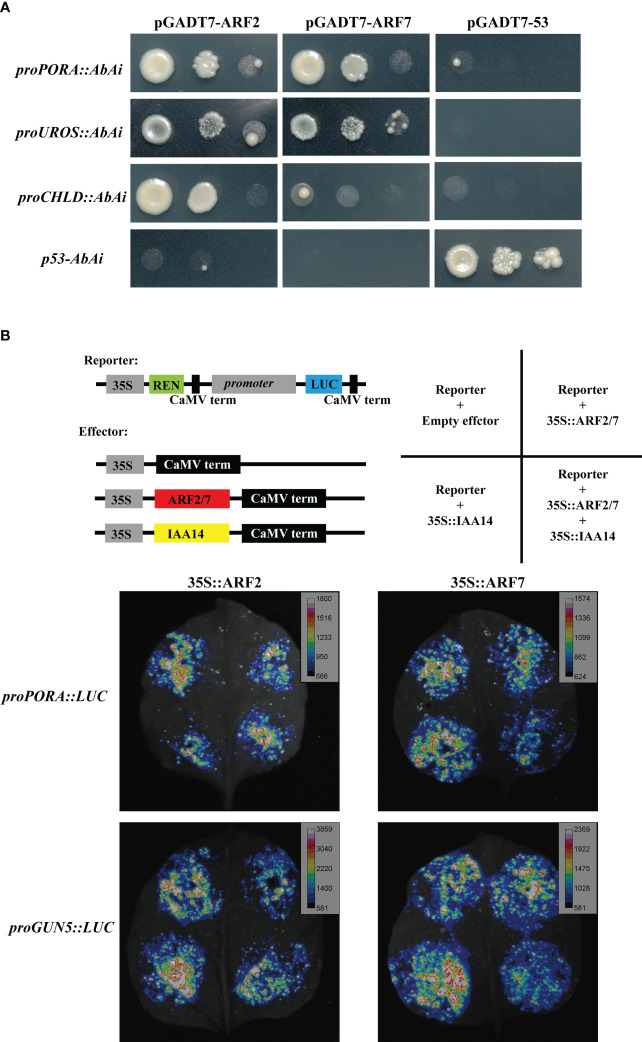
Interaction between ARFs and chlorophyll biosynthesis genes. **(A)** Yeast-one-hybrid assay showing ARF2 and ARF7 bind to the promoter of *PORA*, *UROS* and *CHLD*; **(B)** *proPORA* and *proGUN5* were suppressed by ARF2 and ARF7 cooperated with IAA14 in luciferase reporter assays.

Yeast-one-hybrid assay was first performed to test the interaction between ARFs and chlorophyll biosynthesis gene promoters. Three promoters, *proPORA*, *proUROS* and *proCHLD* were proved to be bound by ARF2 and ARF7, although the interaction between *proCHLD* and ARF7 was weak (**Figure 8A**). The binding of ARF2 and ARF7 to the promoter of *GUN5* was not sure, due to the self-activation of *proGUN5* in the control group (**Figure S5**). Next, luciferase reporter assays were performed in tobacco (*Nicotiana benthamiana*) leaves to confirm the binding of ARFs to the promoter of chlorophyll biosynthesis gene promoters in planta. Considering that ARF7 is one targets of IAA14 (Fukaki et al., 2005; Okushima et al., 2005; Fukaki et al., 2006), which also plays an important role in repressing detached root greening (Kobayashi et al., 2012), we included IAA14 as a corepressor in the luciferase assay. Compared with the control group, the luciferase activities driven by *proPORA*, *proGUN5* and *proCHLD* were significantly repressed when co-expressed with ARF7 alone or ARF7 cooperated with IAA14, but the inhibition was not obvious when co-expressed with ARF2 and IAA14 (**Figure 8B**, **Figure S4**). As for *proUROS::LUC*, the luciferase activity was repressed significantly by both ARF2 and ARF7 and further decreased when co-expression with IAA14 (**Figure S6**). Taken together, these results indicated that ARF2 and ARF7 directly bound to the promoter of chlorophyll biosynthesis genes promoter to suppress their expression.

## Discussion

The chlorophyll biosynthesis process is strictly organized and regulated, any mutation or misregulation of chlorophyll biosynthesis genes will result in abnormal chlorophyll content and changed leaf color. Here, we showed that auxin-defective *yuc2 yuc6* mutant exhibited greener leaves with higher chlorophyll content than WT, and exogenous IAA treatment led to a significant reduction in chlorophyll accumulation in etiolated seedlings, indicating that auxin plays a negative role in regulating chlorophyll biosynthesis. In fact, there are several controversial results between previous reports and our study. First, many researchers reported that auxin could induce the production of chlorophyll (Hunt et al., 2011), but our study demonstrated the direct inhibition of auxin on chlorophyll accumulation and gene expression during de-etiolation. This could be attributed to the difference among species or tissues treated with auxin, because auxin acts as a double-edged sword, and different species or tissues may have variable sensitivity to auxin. Second, alteration of chlorophyll was an indirectly outcome of auxin treatment in some of previous reports (Hunt et al., 2011). In our study, we have showed the inhibitory effect of auxin on chlorophyll synthesis by supplying IAA in MS medium during seedling de-etiolation. In addition, the *GUS* activity driven by *PORA* and *GUN5* promoters was decreased significantly under IAA treatment, implying the direct repression of auxin on *PORA* and *GUN5* expression and chlorophyll synthesis. Together with the reduction in chlorophyll accumulation and the expression of chlorophyll biosynthesis genes in auxin-defective mutants, these results demonstrated that auxin functioned directly as a negative regulator in chlorophyll biosynthesis. Most importantly, the mechanisms revealed previously were mainly focused on the upstream regulators in auxin signaling, such as GOLDEN1/2-LIKE (GLKs) and LONG HYPOCOTYL5 (HY5) (Sagar et al., 2013), but our research draw attention on the immediate interplay between auxin signaling and chlorophyll biosynthesis genes.

The link between auxin and chloroplast development has been highlighted in a number of previous studies (Liu et al., 2020; Salazar-Iribe and De-la-Pena, 2020). There are clues providing how auxin could be directly or indirectly involved in the development of chloroplasts, e.g., by modifying the expression of *GLK2*, *HY5* or *SHIKIMATE KINASE-LIKE* (*SKL*) genes (Kobayashi et al., 2017; Xu et al., 2018; Duarte Lupi et al., 2019). Many genes have been identified to regulate chloroplast development, as the mutants of these genes display altered chloroplast structure, such as auxin signaling-related genes including *IAA/AUXs*, *ARF*s, *GH3*, *SAUR* and *TIR1* (Arsovski et al., 2018; Cruz et al., 2018; Zou et al., 2020) auxin biosynthesis-related genes *YUCCA* and auxin transport-related genes *PIN*. (Chen et al., 2015; Zheng et al., 2016). In this study, the ultrastructure of chloroplasts in *yuc2 yuc6* differed from that in WT, providing an direct evidence of the role of auxin in chloroplast development. Furthermore, the chlorophyll biosynthesis and photosynthesis genes were tight co-expressed, indicating that the signaling governing chlorophyll metabolism also regulated the assembly of the photosynthesis machinery. Our research also confirmed that photosynthesis was improved in *yuc2 yuc6*, suggesting negatively regulation of auxin on photosynthetic activity. Higher accumulation of starch grains was observed in *yuc2 yuc6* chloroplast as compared to WT, which may be resulted from the disintegration of chloroplast in advance because *yuc2 yuc6* grew a little faster than WT. However, the size of leaves and the average chloroplast number per mesophyll cell in *yuc2 yuc6* mutant were larger than those in WT, which may be the reason why *yuc2 yuc6* mutant showed slightly higher photosynthetic rate as compared to WT. Besides, the increased expression of *CABs* may partially explain the improved photochemical efficiency in *yuc2 yuc6*, as *CABs* encode chlorophyll binding proteins associated with chlorophyll and xanthophylls, which absorb sunlight and transfer the excitation energy to the PSII core complexes to drive photosynthetic electron transport (Larouche et al., 1991; Pichersky et al., 1991).

Auxin signaling transduction *via* the action of transcription factors ARF has been verified to play critical roles in regulating chlorophyll biosynthesis by directly or indirectly interaction with chlorophyll biosynthesis genes in tomato (Sagar et al., 2013; Yuan et al., 2018; Yuan et al., 2019). In our research, we demonstrated that ARF2 and ARF7 could directly bind to chlorophyll gene promoters and regulate their expression. More importantly, the concerted function of IAA14 was verified. In addition, in previous study, detached roots cultured *in vitro* can develop chloroplasts and synthesize chlorophyll. In this process, *IAA14* and *ARF7/19* play important roles in inhibiting chlorophyll synthesis in detached root, but *arf7 arf19* (double loss-of-function mutant of *ARF7* and *ARF19*) is less pronounced than *slr-1* (gain-off function mutant of *IAA14*), indicating that other ARFs potentially targeted by IAA14 may negatively regulate chlorophyll biosynthesis in the root (Kobayashi et al., 2012). Our results implied that IAA14 could strengthen the repression of transcription activity of ARF2 as well. On the other hand, ARFs function as either transcriptional activators or repressors. In tomato, SlARF4 negatively regulates chlorophyll accumulation and starch biosynthesis (Sagar et al., 2013), and SlARF10 positively regulated chlorophyll accumulation *via* direct activation of the expression of *SlGLK1* (Yuan et al., 2018), while SlARF6A positively regulates the expression of *SlGLK1*, *CAB*, and *RbcS* and negatively regulated *SAMS1* expression at the same time (Yuan et al., 2019). Whether other ARFs and AUX/IAAs in *Arabidopsis* involved in regulating chlorophyll accumulation and the underlying mechanisms demand further investigation.

## Data availability statement

The original contributions presented in the study are included in the article/**Supplementary Material**. Further inquiries can be directed to the corresponding authors.

## Author contributions

L-TX, YY, and W-GL conceived the research. W-GL and Q-WL performed the experiments. W-GL, YS, CH, and B-XM analyzed the data. W-GL and YY wrote the manuscript. L-TX supervised the project and revised the manuscript. All authors contributed to the article and approved the submitted version.
