# Supplementary material for: Auxin inhibits chlorophyll accumulation through ARF7-IAA14-mediated repression of chlorophyll biosynthesis genes in Arabidopsis
Source: Front Plant Sci. 2023 Apr 20;14:1172059. doi: 10.3389/fpls.2023.1172059 (PMC10157223; doi:10.3389/fpls.2023.1172059)
Supplement: Supplementary file 1 [file DataSheet_1.docx]

Supplementary Material

**Auxin inhibits chlorophyll accumulation through ARF7-IAA14-mediated repression of chlorophyll biosynthesis genes in Arabidopsis**

**Wei-Gui Luo, Qi-Wen Liang, Yi Su, Chao Huang, Bei-Xin Mo, Yu Yu^*^, Lang-Tao Xiao^*^**

*** Correspondence:** Lang-Tao Xiao: [ltxiao@hunau.edu.cn](mailto:ltxiao@hunau.edu.cn); Yu Yu: [yuy@szu.edu.cn](mailto:yuy@szu.edu.cn).


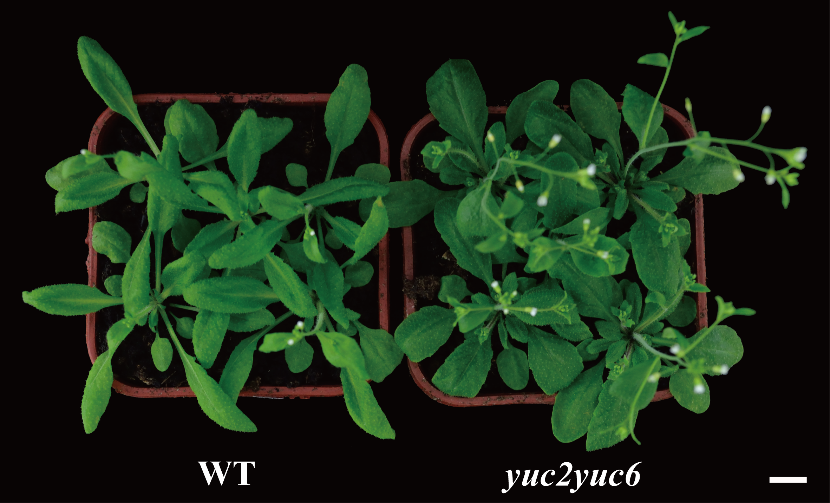

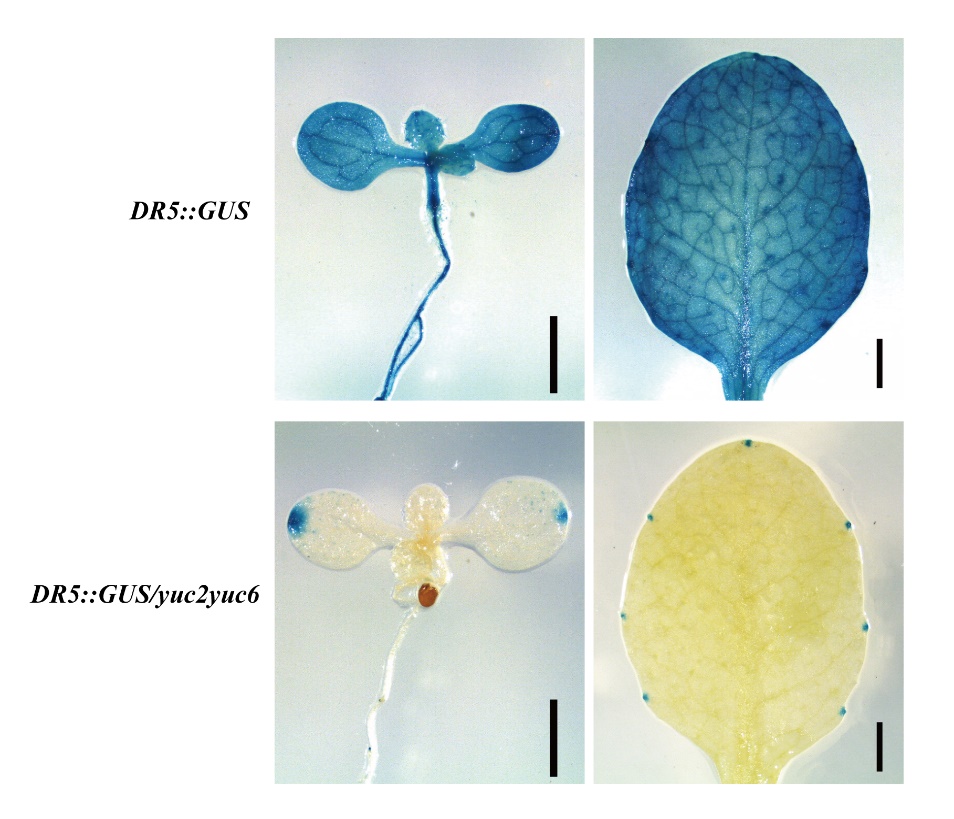
**Supplementary Figure 1.** Phenotypes of WT and *yuc2 yuc6* mutant. Bar = 1 cm.

**Supplementary Figure 2.** GUS staining of *DR5::GUS* in WT and *yuc2 yuc6* mutant. Bar = 2 mm.


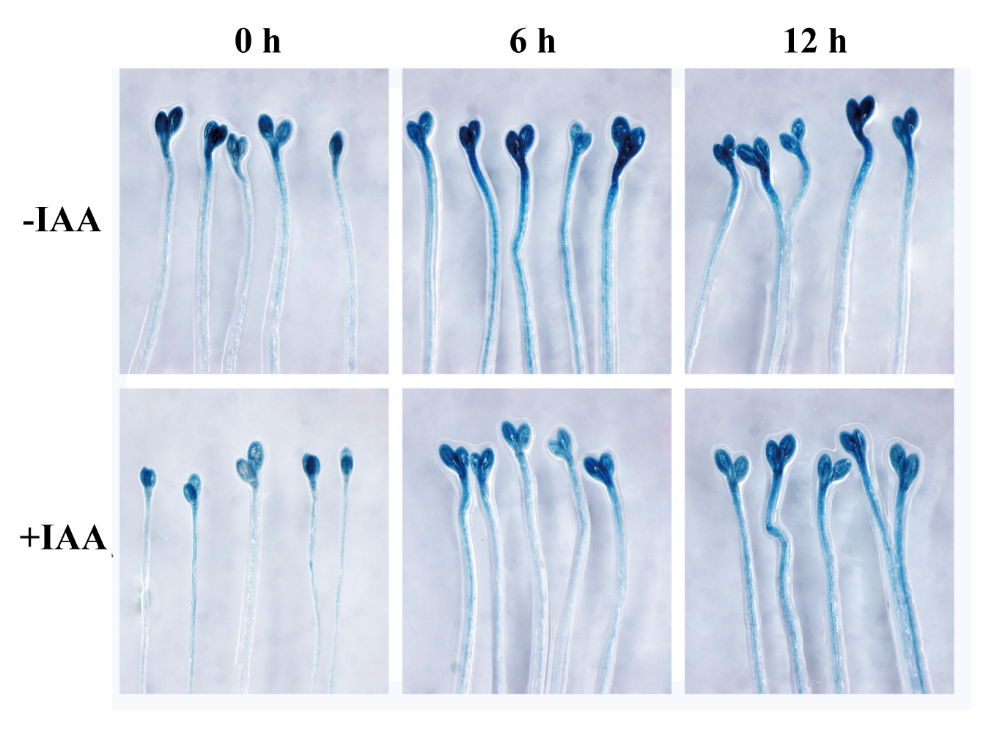

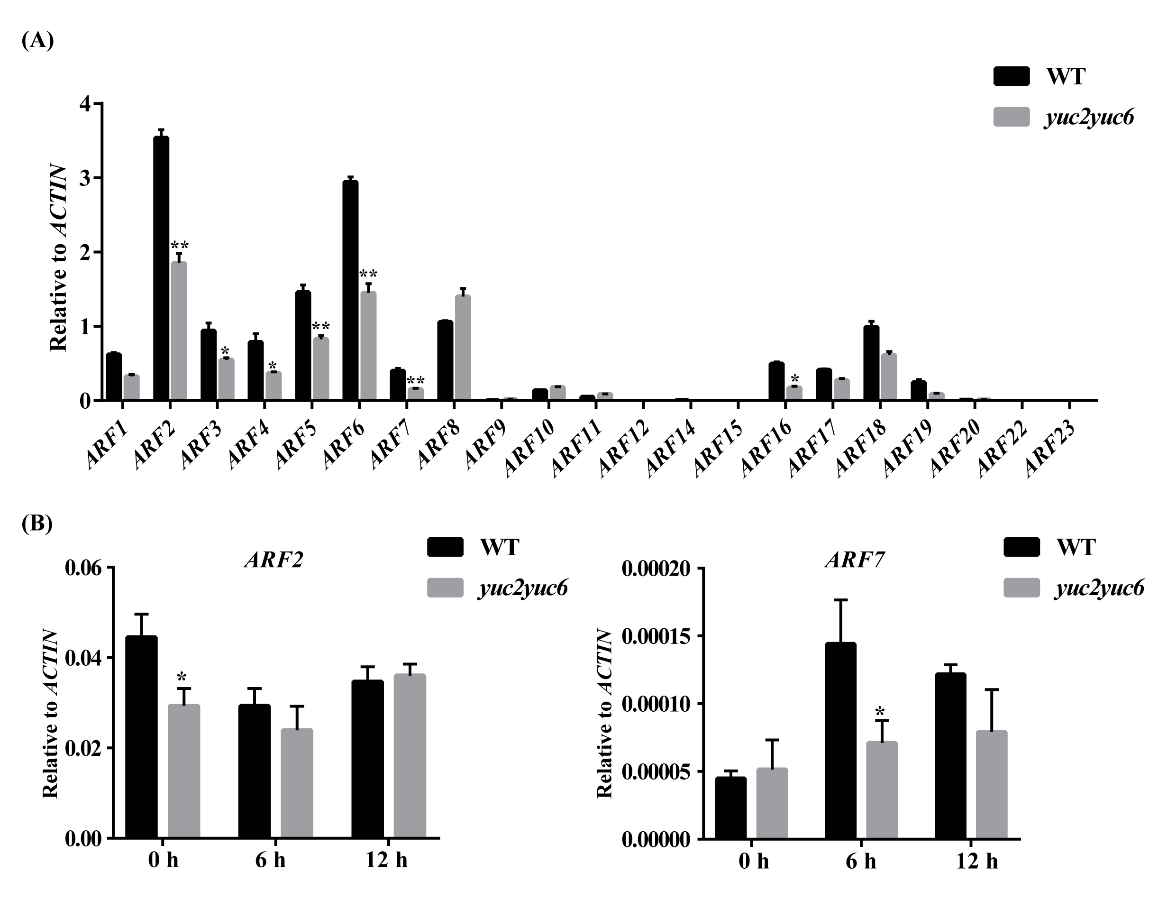
**Supplementary Figure 3.** Auxin suppresses the expression of *GUS* driven by *GUN5* promoter.

**Supplementary Figure 4.** Expression levels of *ARF* genes in WT and *yuc2 yuc6*. (A) Transcript levels of *ARF* genes in rosette leaves; (B) The expression levels of *ARF2* and *ARF7* in seedlings during de-etiolation. * represents *p* value <0.05, ** represents *p* value <0.01.


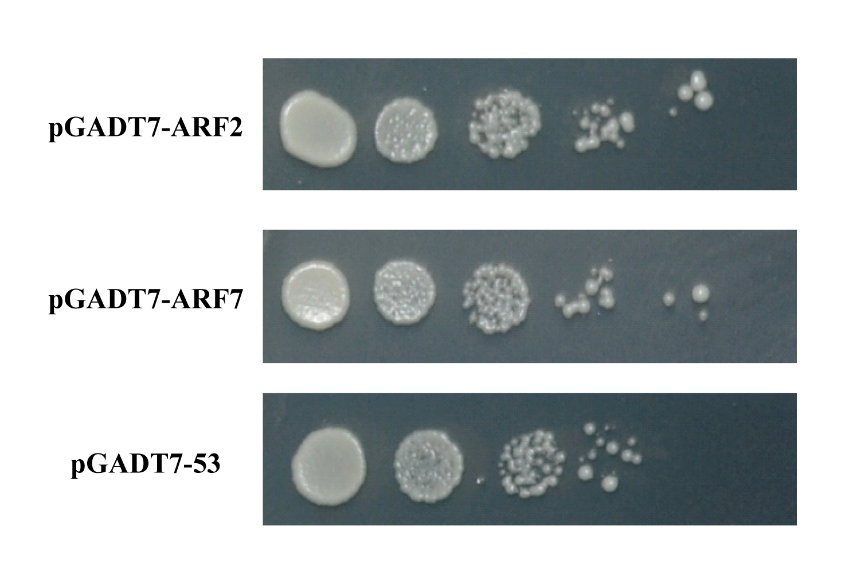

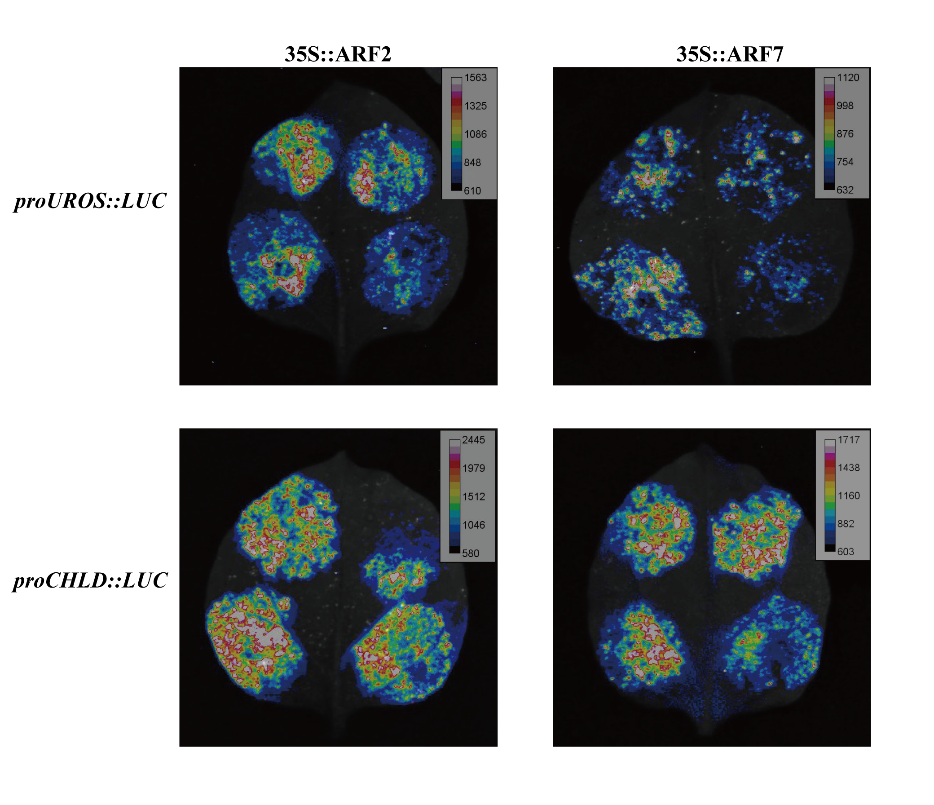
**Supplementary Figure 5.** Yeast-one-hybrid assay of *proGUN5* and ARFs.

**Supplementary Figure 6.** The activities of *proUROS* and *proCHLD* were suppressed by ARF2 and ARF7 cooperated with IAA14 in luciferase reporter assays.
