# Supplementary material for: Auxin inhibits chlorophyll accumulation through ARF7-IAA14-mediated repression of chlorophyll biosynthesis genes in Arabidopsis
Source: Front Plant Sci. 2023 Apr 20;14:1172059. doi: 10.3389/fpls.2023.1172059 (PMC10157223; doi:10.3389/fpls.2023.1172059)
Supplement: Supplementary file 2 [file Table_1.docx]

Table S1. *AuxREs* in the promoters of chlorophyll biosynthesis genes

| Locus | Gene name | Position | Strand | Sequence |
| --- | --- | --- | --- | --- |
| AT1G58290 | *HEMA1* | -1669  -1245  270 | -  -  + | gAGACA  gAGACA  TGTCTc |
| AT5G63570 | *GSA1* | 134  157 | -  - | gAGACA  gaTGTCGgcg |
| AT2G26540 | *UROS* | -1133  -438  -37  103 | +  -  -  - | TGTCTc  gAGACA  gAGACA  gAGACA |
| AT4G01690 | *PPO1* | -648 | - | gAGACA |
| AT5G45930 | *CHLI2* | -379 | - | gAGACA |
| AT1G08520 | *CHLD* | -1627  -1087  -5  51 | -  -  -  - | gAGACA  gAGACA  gAGACA  gAGACA |
| AT5G13630 | *GUN5* | -999  -948  264  88 | -  +  -  + | gAGACA  TGTCTc  gAGACA  atcCGACAaa |
| AT5G54190 | *PORA* | -441  -42 | +  + | TGTCTc  TGTCTc |
| AT3G51820 | *CHLG* | -1837  -935 | +  + | TGTCTc  TGTCTc |
| AT1G29930 | *CAB1* | -941 | - | gAGACA |
| AT1G29920 | *CAB2* | -980 | + | TGTCTc |
| AT1G29910 | *CAB3* | -1530 | - | gAGACA |

Note: Position refer to the distance to Transcription Star Site; + represents the sense strand, - represents the antisense strand.

Table S2. Primers used for vector construction

| DNA | Primer sequence | vector |
| --- | --- | --- |
| *proPORA* | CTATGACCATGATTACGAATTCGTAAAGGGTGCATTGGTTGG | pCAMBIA1301 |
|  | TCAGATCTACCATGGTGTTTCGTTTAAGACTTAAAG |  |
| *proGUN5* | CTATGACCATGATTACGAATTCATCTGTCACTTGAAATTAAACC | pCAMBIA1301 |
|  | TCAGATCTACCATGGTTTGCGGCTGCTGGATTCTCC |  |
| *proPORA* | AAATGATGAATTGAAAAGCTTCCAATATAGTTTGCTTCGATG | pAbAi |
|  | ATACAGAGCACATGCCTCGAGTGTTTCGTTTAAGACTTAAAG |  |
| *proGUN5* | AAATGATGAATTGAAAAGCTTATCTGTCACTTGAAATTAAACC | pAbAi |
|  | ATACAGAGCACATGCCTCGAGGCACTCCACCCAAGAAAACGCAG |  |
| *proUROS* | AAATGATGAATTGAAAAGCTTATATCTAGGTTTTAAAGTTAACAG | pAbAi |
|  | TACAGAGCACATGCCTCGAGTAGAAAAAGTTCCAAGATGGAGAG |  |
| *proCHLD* | AAATGATGAATTGAAAAGCTTATATATGTGGATATGAGAGTCGG | pAbAi |
|  | ATACAGAGCACATGCCTCGAGAGCACTATCCACATTAAGATTTC |  |
| *ARF2* CDs | GCCATGGAGGCCAGTGAATTCATGGCGAGTTCGGAGG | pGADT7 |
|  | CAGCTCGAGCTCGATGGATCCTTAAGAGTTCCCAGCGC |  |
| *ARF7 CDs* | GCCATGGAGGCCAGTGAATTCATGAAAGCTCCTTCATC | pGADT7 |
|  | CAGCTCGAGCTCGATGGATCCTCACCGGTTAAACGAAG |  |
| *proPORA* | TTCCTGCAGCCCGGGGGATCC GTAAAGGGTGCATTGGTTGGTTC | pGreenII 0800-LUC |
|  | CGCTCTAGAACTAGTGGATCC TGTTTCGTTTAAGACTTAAAGAG |  |
| *proGUN5* | TTCCTGCAGCCCGGGGGATCC GAGTAAGACAGACAAACATGG | pGreenII 0800-LUC |
|  | CGCTCTAGAACTAGTGGATCC TTTGCGGCTGCTGGATTCTC |  |
| *proUROS* | TTCCTGCAGCCCGGGGGATCC CAATACCCAGGAGAAAACATTGG | pGreenII 0800-LUC |
|  | CGCTCTAGAACTAGTGGATCCTAGAAAAAGTTCCAAGATGGAGAG |  |
| *proCHLD* | TTCCTGCAGCCCGGGGGATCCACAAGATTGGTTAACCTAATTAGG | pGreenII 0800-LUC |
|  | CGCTCTAGAACTAGTGGATCCTTTCAAAGATAGGAGAAGAAGAAG |  |
| *ARF2* CDs | CGCTCTAGAACTAGTGGATCC ATGGCGAGTTCGGAGGTTTC | pGreenII 62-SK |
|  | GTCGACGGTATCGATAAGCTT TTAAGAGTTCCCAGCGCTGG |  |
| *ARF7* CDs | CGCTCTAGAACTAGTGGATCC ATGAAAGCTCCTTCATCAAATGG | pGreenII 62-SK |
|  | GTCGACGGTATCGATAAGCTT TCACCGGTTAAACGAAGTGG |  |
| *IAA14* CDs | CGCTCTAGAACTAGTGGATCC ATGAACCTTAAGGAGACGGAGC | pGreenII 62-SK |
|  | GTCGACGGTATCGATAAGCTT TTATTGGAAAAACAGAAAAGAGCC |  |

Table S3. Primers used for qPCR

| Gene | Primer name | Primer sequence |
| --- | --- | --- |
| *HEMA* | qHEMA1-F | GAGTGGCAGAGCTCGAGAAG |
|  | qHEMA1-R | TCGCTCAGCGTTCTACTGTC |
| *GSA1* | qGSA1-F | GGAACTAACCAACGGAATCT |
|  | qGSA1-R | CTCTGTGTCGCTCTTCTTTG |
| *UROS* | qUROS-F | CTTCTGCAGTTCGCGCCT |
|  | qUROS-R | TATGCTCTCGACCCACCCTT |
| *PPO1* | qPPO1-F | CCCGAAAGAAGCAATCCGAA |
|  | qPPO1-R | AGACCCGCCAATGTAGTTCA |
| *CHLI2* | qCHLI2-F | TCCTGCTCGGTTTATCCTCA |
|  | qCHLI2-R | CGTCTCTAACCGTCCCTACT |
| *CHLD* | qCHLD-F | AGCCAATGCTTCCAAAGGGT |
|  | qCHLD-R | CCTTGCCATTCTTTTGGCCC |
| *GUN5* | qGUN5-F | TGGAAGAGTGAACCGTGTCG |
|  | qGUN5-R | AGATCACGGAAGACCCCTGA |
| *PORA* | qPORA-F | TGAGATGCAAGAGGGAACAGAG |
|  | qPORA-R | AAGAAGCTCCCGTGACAAC |
| *CHLG* | qCHLG-F | CACTGTCTCCACCATCCACT |
|  | qCHLG-R | ATCAGTATCAGTCTCCGCCG |
| *CAB1* | qCAB1-F | GCCGGTAAGGCCGTCAA |
|  | qCAB1-R | GGCTTGGCAACAGTCTTCCT |
| *CAB2* | qCAB2-F | TGGAGAGGCAGTTTGGTTCA |
|  | qCAB2-R | CTGTAACCTTCAACGGCTCC |
| *CAB3* | qCAB3-F | GGCTACAGAGTCGCAGGAAA |
|  | qCAB3-R | GAAAGCCTCTGGGTCGGTAG |
| *ACTIN2* | qACTIN-F | AAGTCTTGTTCCAGCCCTCG |
|  | qACTIN-R | TTTGCTCATACGGTCAGCGA |
